# Cellular Immunity in COVID-19 Convalescents with PCR-Confirmed Infection but with Undetectable SARS-CoV-2–Specific IgG

**DOI:** 10.3201/eid2701.203772

**Published:** 2021-01

**Authors:** Sina Schwarzkopf, Adalbert Krawczyk, Dietmar Knop, Hannes Klump, Andreas Heinold, Falko M. Heinemann, Laura Thümmler, Christian Temme, Marianne Breyer, Oliver Witzke, Ulf Dittmer, Veronika Lenz, Peter A. Horn, Monika Lindemann

**Affiliations:** Institute for Transfusion Medicine, University Hospital Essen, University of Duisburg-Essen, Essen, Germany (S. Schwarzkopf, D. Knop, H. Klump, A. Heinold, F.M. Heinemann, L. Thümmler, C. Temme, M. Breyer, V. Lenz, P.A. Horn, M. Lindemann);; Department of Infectious Diseases, West German Centre of Infectious Diseases, Universitätsmedizin Essen, University of Duisburg-Essen, Essen (A. Krawczyk, O. Witzke);; Institute for Virology, University Hospital Essen, University of Duisburg-Essen, Essen (U. Dittmer)

**Keywords:** coronavirus disease, SARS-CoV-2, severe acute respiratory syndrome coronavirus 2, severe acute respiratory syndrome, SARS, viruses, respiratory infections, zoonoses, COVID-19, PCR-confirmed infection, seronegativity, cellular immunity, ELISpot, convalescent plasma, T cells, B cells

## Abstract

We investigated immune responses against severe acute respiratory syndrome coronavirus 2 (SARS-CoV-2) among a group of convalescent, potential blood donors in Germany who had PCR-confirmed SARS-CoV-2 infection. Sixty days after onset of symptoms, 13/78 (17%) study participants had borderline or negative results to an ELISA detecting IgG against the S1 protein of SARS-CoV-2. We analyzed participants with PCR-confirmed infection who had strong antibody responses (ratio >3) as positive controls and participants without symptoms of SARS-CoV-2 infection and without household contact with infected patients as negative controls. Using interferon-γ ELISpot, we observed that 78% of PCR-positive volunteers with undetectable antibodies showed T cell immunity against SARS-CoV-2. We observed a similar frequency (80%) of T-cell immunity in convalescent donors with strong antibody responses but did not detect immunity in negative controls. We concluded that, in convalescent patients with undetectable SARS-CoV-2 IgG, immunity may be mediated through T cells.

One promising therapeutic option to treat severely ill patients with coronavirus disease (COVID-19) is the use of convalescent plasma (CP) of donors who recovered from severe acute respiratory syndrome coronavirus 2 (SARS-CoV-2) infection (*1*–*3*). When searching for potential CP donors with PCR-confirmed SARS-CoV-2 infection, we observed that 17% of those who volunteered had either borderline or negative results (ratio <1.1) to a SARS-CoV-2 IgG ELISA. We decided to follow up with these volunteers by repeating antibody tests and by assessing T-cell immunity by enzyme-linked immunospot (ELISpot) assay for interferon-γ (IFN-γ).

Zhao et al. described the importance of T cells for the recovery from a structurally related coronavirus, the Middle East respiratory syndrome (MERS) virus, in 2017 (*4*); Braun et al. speculated that T-cell immunity could also be protective against infection with SARS-CoV-2 (*5*). Recently, SARS-CoV-2–specific T cells were detected in persons with a history of mild COVID-19 infection and in antibody-seronegative family members of COVID-19 patients (*6*; F. Gallais et al., unpub. data. https://doi.org/10.1101/2020.06.21.20132449). However, the seronegative persons in these 2 previous studies either tested negative or were not tested by SARS-CoV-2 PCR. It remains unclear if the T-cell responses detected in SARS-CoV-2 IgG–negative persons without PCR-confirmed infection were induced by SARS-CoV-2 or prior infection with a different coronavirus. In our study, we focused on a cohort with mild PCR-confirmed SARS-CoV-2 infection and undetectable SARS-CoV-2 IgG. This group is unique because it is biased toward male volunteers who felt healthy enough to donate blood.

In this study we established a SARS-CoV-2–specific ELISpot assay and analyzed the T-cell responses in distinct groups of potential blood donors; donors with a previously PCR-confirmed SARS-CoV-2 infection and undetectable or strong spike S1 IgG response; and SARS-CoV-2–negative controls without a history of COVID-19–related symptoms or household contact with infected patients. We furthermore conducted follow-up testing for immunity to SARS-CoV-2 until a median of 75 days (range 24–154) after the onset of symptoms and correlated results of SARS-CoV-2–specific T- and B-cell immunity. 

The study was approved by the local ethics committee (approval no. 20-9225-BO). All volunteers provided informed consent to participate in the study, which has been performed in accordance with the ethics standards noted in the 1964 Declaration of Helsinki and its later amendments or comparable ethics standards.

## Materials and Methods

### Study Participants

We used a questionnaire to look for potential plasma donors who recovered from SARS-CoV-2 infection. The questionnaire addressed characteristics and additional parameters determining suitability as blood donor (Table). We received >550 questionnaires; 310 volunteers had had PCR-confirmed SARS-CoV-2 infection. We selected 78 volunteers (54 male, 24 female) who had PCR-confirmed SARS-CoV-2 infection as participants. Their median age was 47 years (19–66) years. We preferred donors who tested negative by a second SARS-CoV-2 PCR and had experience donating blood; we especially sought donors with the blood type AB (because they are universal plasma donors) and donors with a body weight >60 kg (because they can donate 3 units of convalescent plasma). We preferentially recruited those living in close vicinity to University Hospital Essen. Because we observed antibodies against human leukocyte antigens and human neutrophil antigens, which prohibited blood donation, mainly in female patients (n = 4), we preferred male donors, of whom 1 had these leukocyte antibodies. Only 4 of the participants received oxygen supplementation and none ventilator treatment. Of note, 39/78 participants were infected during skiing holidays. Unfortunately, radiograph and computed tomography data were not available; they are usually performed only for critically ill patients in Germany. Thus, our cohort is unique because it is biased toward especially healthy male blood donors with mild courses of COVID-19. We tested donor serum samples for IgG against the S1 protein of SARS-CoV-2 by ELISA. Furthermore, results of a standard neutralization assay were available in donors with negative or borderline antibody ratios.

**Table:** Clinical characteristics of 78 potential convalescent-plasma donors with PCR-confirmed severe acute respiratory syndrome coronavirus 2 infection, grouped by antibody ratio, Germany*

Parameter	Ratio <1.1	Ratio 1.1–3.0	Ratio >3.0
All donors	13 (16.7)	24 (30.8)	41 (52.6)
Age, y	45 (19–55)	51 (28–65)	47 (20–66)
Sex, M/F	6/7	19/5	29/12
Body mass index	25.2 (22.3–30.1)	24.9 (22.8–28.1)	26.7 (22.2–36.8)
Interval to onset of symptoms, d	52 (32–100)	60 (24–98)	64 (22–112)
Stay in risk area/risk contact	6	17	20
Symptoms of COVID-19 infection			
Cough	7	19	30
Fever	9	13	24
Shortage of air	3	4	15
Rhinitis	7	7	16
Sore throat	6	8	15
Limb pain	7	15	19
Shivering	6	8	15
Diarrhea	5	6	15
Weight loss	3	2	9
Nausea	0	0	4
Loss of appetite	5	3	12
Headaches	7	16	26
Skin rash	1	1	1
Swelling of lymph nodes	3	0	2
Loss of sense of smell and taste	8	16	18
Necessity of oxygen supply	0	1	3
Antimicrobial treatment	1	3	7
Blood group			
O	5	12	16
A	6	8	16
B	0	0	6
AB	1	3	3
ND	1	1	0

*Values are no. donors or median (range). Ratio is IgG against the S1 protein. ND, not determined.

In the negative control group we included 22 healthy participants (6 male, 16 female) who had no symptoms of SARS-CoV-2 infection and no household contact with infected patients since January 2020. Their median age was 48 years (range 28–60 years).

### Antibody ELISA

We determined IgG antibodies by a CE-marked Anti-SARS-CoV-2 IgG semiquantitative ELISA (Euroimmun, https://www.euroimmun.com), according to the manufacturer’s instructions. The ELISA plates were coated with recombinant SARS-CoV-2 spike protein (S1 domain). Serum samples were analyzed automatically at 1:100 dilution, using the Immunomat (Virion\Serion, https://www.virion-serion.de). Results are given as the ratio of patient sample/control sample). An antibody ratio of >1.1 was considered positive, of ≥0.8 to <1.1 borderline, and of <0.8 negative.

### Virus Neutralization Assay

Vero E6 cells (ATCC CRL-1586, https://www.atcc.org) and SARS-CoV-2 virus were cultured as described by Heilingloh et al. (*7*). Neutralization capacity of serum samples was determined by endpoint dilution assay, expressed as 50% tissue culture infective dose (TCID_50_)/mL. Serial dilutions (1:20–1:2560) of serum samples were incubated with 100 TCID_50_ of SARS-CoV-2 for 1 h at 37°C and added afterwards to confluent Vero E6 cells cultured in 96-well microtiter plates. On day 3 after infection, the cells were stained with crystal violet (Roth, https://www.carlroth.com) and dissolved in 20% methanol (Merck, https://www.merck.com); we analyzed the appearance of cytopathic effects (CPE) by light microscopy. The neutralizing titer was defined as the reciprocal of the highest serum dilution at which no CPE breakthrough in any of the triplicate cultures was observed.

### ELISpot Assay

ELISpot stripes containing PVDF membranes (MilliporeSigma MultiScreen HTS; Fisher Scientific, https://www.fishersci.com) were activated with 50 µL of 35% ethanol for 10 s and washed with distilled water. Plates were then coated for 3 h with 60 µL of monoclonal antibodies against IFN-γ (10 µg/mL of clone 1-D1K; Mabtech, https://www.mabtech.com). Thereafter, ELISpot plates were washed and then blocked with 150 µL AIM-V (Thermo Fisher Scientific, https://www.thermofisher.com). After 30 min at 37°C, AIM-V was discarded and duplicates of 250,000 peripheral blood mononuclear cells (PBMC) were grown in the presence or absence of either PepTivator SARS-CoV-2 protein S1/S2 or membrane (M) protein (600 pmol/mL of each peptide; Miltenyi Biotec, https://www.miltenyibiotec.com) or an S1 protein (4 µg/mL; Sino Biologic, https://www.sinobiological.com) in 150 µL of AIM-V. The peptide mix (PepTivator) of the S1/S2 protein covered the immunodominant domains, the peptide mix of the M protein the complete sequence of the glycoprotein. The S1 protein was a recombinant protein expressed in (human) HEK293 cells (Appendix Figure 1). After 19 h incubation at 37°C, the ELISpot plates were washed and captured IFN-γ was detected by incubation for 1 h with 50 µL of the alkaline phosphatase-conjugated monoclonal antibody against IFN-γ (clone 7-B6–1, Mabtech), diluted 1:200 with phosphate-buffered saline plus 0.5% bovine serum album. After further washing, 50 µL of nitro blue tetrazolium/5-bromo-4-chloro-3-indolyl-phosphate was added; purple spots appeared within 7 min. Spot numbers were analyzed by an ELISpot reader (AID Fluorospot, Autoimmun Diagnostika GmbH, https://www.aid-diagnostika.com). Mean values of duplicate cell cultures were considered. We determined SARS-CoV-2–specific spots by spot increment, defined as stimulated minus nonstimulated values. Stimulated spot numbers >3-fold higher than negative (unstimulated) controls combined with an increment value of >3 to any of the 3 antigens were considered positive. Of note, the negative controls reached a mean value of 0.27 spots and an SD of 0.48 (Appendix).

### Statistical Analysis

We performed statistical analysis using GraphPad Prism version 8.0.1 (https://www.graphpad.com) and IBM SPSS Statistics 23 (https://www.ibm.com/spss/statistics) software. We used linear regression analysis for numerical variables. The analysis of categorical variables was performed by Mann-Whitney test or 1-way analysis of variance (Kruskal-Wallis test) with Dunn’s correction for multiple comparisons, as appropriate. Two-sided p values <0.05 were considered significant.

## Results

**Figure 1:**
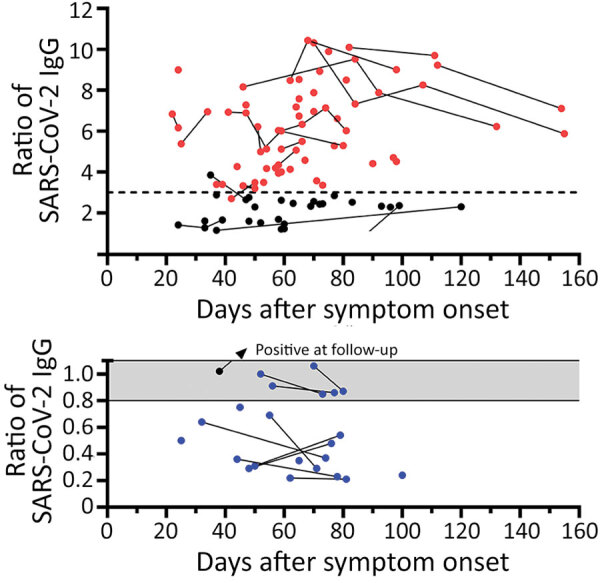
Distribution of SARS-CoV-2 IgG in 78 potential convalescent-plasma donors with PCR-confirmed infection, Germany. Red dots represent study participants with antibody ratio >3; black dots, participants with a ratio of 1.1–3; blue dots, participants with ratio <1.1. Sequential data are connected. Horizontal dashed line indicates a ratio of 3. A) Antibody ratios in the positive or intermediate range. B) Antibody ratios in the borderline or negative range. Gray shading indicates borderline values (ratio of 0.8–1.1); scale is adjusted to enhance data visualization. SARS-CoV-2, severe acute respiratory syndrome coronavirus 2.

In 78 potential convalescent plasma donors with PCR-confirmed SARS-CoV-2 infection, the median interval between onset of symptoms and first blood sampling was 60 days (range 22–112 days) (Figure 1). Thirteen out of 78 (17%) donors had either borderline or negative results (ratio <1.1) to the Anti-SARS-CoV-2 IgG ELISA (Euroimmun). Altogether, 28 CP donors were tested again at later time points, a median of 75 days (24–154 days) after onset of symptoms. Retesting in 10 participants with a ratio of <1.1 showed that most antibody results (9/10) remained similar. The median interval between both blood samplings in this group was 25 days (range 10–61 days). In 1 volunteer with a borderline ratio, the value increased over time and became positive. In donors with higher antibody ratios, the values also remained at a similar level. In all participants with an antibody ratio <0.8, no neutralizing antibodies could be found; in those with a ratio of 0.8–1.1, the titer was 1:20–1:40.

We compared the characteristics of participants with undetectable antibodies to those with intermediate (1.1–3.0) or high antibody ratio (>3) (Table). Of note, in the total cohort of 78 potential blood donors, the median antibody ratio was 3.37; we chose a ratio of 3 as our internal cutoff for convalescent plasma donations. None of the parameters, including age, sex, body mass index, interval to onset of symptoms, risk exposure, symptoms of SARS-CoV-2 infection, need for oxygen or antibiotic treatment, or blood group, differed significantly. However, female participants tended to be overrepresented in the group with undetectable antibodies (p = 0.1 by Kruskal-Wallis test). One of the 78 potential CP donors did not report any symptoms of SARS-CoV-2 infection in the questionnaire. This participant showed an antibody ratio of 3.9.

**Figure 2:**
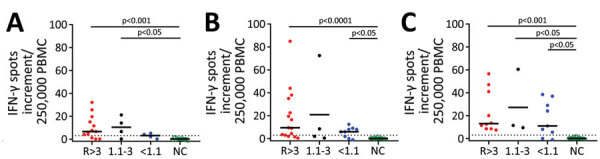
Cellular immunity against severe acute respiratory syndrome coronavirus 2 (SARS-CoV-2) as determined by ELISpot assay in potential convalescent-plasma donors with PCR-confirmed infection, Germany. Peripheral blood mononuclear cells of volunteers were stimulated by an S1 protein antigen of SARS-CoV-2 (A) and by peptide pools of the S1/S2 (B) and the M protein (C). If volunteers were tested sequentially, we only included the first dataset. The 3 left groups represent potential convalescent-plasma donors with PCR-confirmed SARS-CoV-2 infection. They either had a strong positive antibody response to the SARS-CoV-2 IgG ELISA as defined by an antibody ratio (R) of >3 (n = 15), an intermediate response (ratio of 1.1–3, n = 4) or borderline or negative results (ratio of <1.1, n = 9). The right group displays data in healthy controls without symptoms of respiratory or gastrointestinal infections and without household contact with SARS-CoV-2 infected patients since January 2020 (n = 22). The group has tested negative or has not been tested by SARS-CoV-2 PCR. Responses in the 4 groups of volunteers were compared by Kruskal-Wallis test with Dunn’s correction. Dotted lines represent 3 spots increment. Horizontal lines indicate median values. Stimulation by S1 protein could not be performed in 7 volunteers; stimulation by the M peptide pool could not be performed in 6. Red dots represent volunteers with an antibody ratio >3; black dots, volunteers with a ratio of 1.1–3; blue dots, volunteers with ratio <1.1; green, NC. IFN-γ, interferon-γ; NC, negative controls.

**Figure 3:**
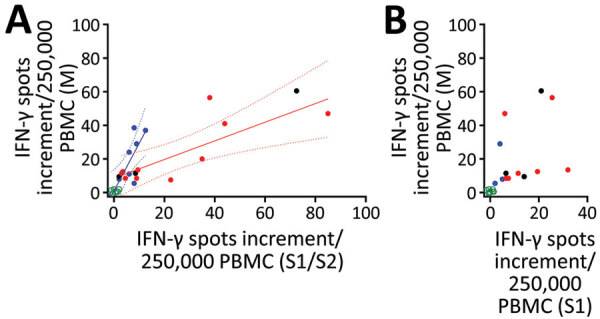
Interrelationship between results of various severe acute respiratory syndrome coronavirus 2 (SARS-CoV-2)–specific cellular assays in 78 potential convalescent-plasma donors with PCR-confirmed infection, Germany. The plots include the first dataset in potential convalescent plasma donors and in negative controls. Red dots represent volunteers with an antibody ratio >3; black dots, volunteers with a ratio of 1.1–3; blue dots, volunteers with ratio <1.1; green dots, NC. PBMCs of volunteers were stimulated by peptide pools of the S1/S2 and the M protein and by an S1 protein antigen of SARS-CoV-2. A) Analysis of ELISpot assay with S1/S2 peptides versus M peptides. We performed 2 linear regression analyses separately for potential plasma donors with an IgG ratio >3 and <1.1. Solid lines show regression lines and dotted lines 95% CI. B) Analysis of ELISpot assay with S1 protein versus M peptides. ELISpot, enzyme-linked immunospot; IFN-γ, interferon-γ; PBMC, peripheral blood mononuclear cells.

Cellular immunity was determined from day 24 to day 154 after the onset of COVID-19 symptoms, parallel to antibody testing (Appendix Figure 2). Immunity was followed up as a control in participants with undetectable or low antibodies (irrespective of a triggering event) or when plasma was donated. We established an IFN-γ ELISpot assay separately for each of various stimuli, peptide pools of the S1/S2 and the M protein, and an S1 protein antigen of SARS-CoV-2 (Figures 2, 3). None of the ELISpot responses differed significantly between 9 participants with undetectable antibody responses (ratio <1.1) and 15 with high antibody responses (ratio >3) (Figure 2). However, ELISpot responses to all stimuli were substantially higher than in the negative controls. Nevertheless, the strength of responses toward the S1 protein tended to be higher in the group with a ratio of >3 versus <1.1 (Figure 2, panel A); whereas it was only marginally higher toward the S1/S2 peptides (Figure 2 panel B) and similar toward M peptides (Figure 2, panel C). The strength of responses toward S1/S2 peptides tended to be higher overall than the S1 protein alone. CP donors with an antibody ratio <1.1 showed a median frequency of 3 spots per 250,000 PBMC for stimulation with S1 protein, 6 with S1/S2 peptides, and 11 with M peptides. CP donors with an antibody ratio >3 showed a median frequency of 7 spots per 250,000 PBMC with S1 protein, 10 with S1/S2 peptides, and 13 with M peptides.

To analyze a possible interrelationship between T cell responses against different SARS-CoV-2 antigens and between T- and B-cell responses, we plotted results of various assays (Figure 3; Appendix Figure 3). We observed that patients whose antibody ratio was <1.1 showed robust ELISpot responses mainly directed against the M protein, whereas patients who had a ratio >3 had responses similarly directed against S1/S2 or S1 protein and M protein (Figure 3). We furthermore found that cellular responses against S1/S2 or S1 protein were all low (maximum of 13 spots increment against S1/S2 and 5 against S1 protein) in participants with an antibody ratio <1.1. In contrast, participants with a ratio >3 reached maximum values of 85 (S1/S2 protein) and 32 (S1 protein) spots increment. Maximum responses toward M peptides were more similar: 39 spots increment in CP donors with a ratio of <1.1 or 57 in those with ratio >3.

Cellular immunity toward any of the SARS-CoV-2 antigens was detectable in 7/9 (78%) participants who had an antibody ratio <1.1. In comparison, 12/15 (80%) donors with an antibody ratio of >3 had detectable cellular immunity. Considering all potential CP donors with PCR-confirmed SARS-CoV-2 infection (also those with a ratio of 1.1–3), 22/28 (79%) were classified as positive by ELISpot.

In summary, we could detect T-cell immunity against SARS-CoV-2 in most of the SARS-CoV-2 PCR-positive healthy participants with undetectable IgG antibodies against the S1 protein. In this group, T-cell immunity was more strongly directed against the M than the S1 protein.

## Discussion

We focused on a cohort of volunteer study participants with PCR-confirmed SARS-CoV-2 infection who did not react positive to an S1-specific SARS-CoV-2 IgG ELISA. We observed undetectable humoral response in 17% of our potential blood donors. Similar to our data, other groups reported a lack of antibody response in a subset of patients infected with SARS-CoV-2. For example, a study in China reported absence of antibodies in 10%–20% of participants (W. Tan et al., unpub. data, https://doi.org/10.1101/2020.03.24.20042382). Moreover, Cervia et al. described that, in 15%–20% of S protein–seronegative patients (IgG in the serum), S protein–specific IgA was detectable at several mucosal sites (C. Cervia et al., unpub. data, https://doi.org/10.1101/2020.05.21.108308). Previous publications demonstrated that the magnitude of the humoral response toward SARS-CoV-2 was dependent on the duration and magnitude of viral antigen exposure (*8**,**9*; C. Cervia et al.). The absence of durable systemic IgG responses may indicate mild and transient SARS-CoV-2 infection that was cleared effectively (e.g., by the innate immune system) (*10*). However, whether this transient immune response had led to protective immunity needs to be clarified. The detection of SARS-CoV-2–specific IgG is not considered consistently to be a correlate of virus control (*11*–*13*).

Protection of humans against reinfection can be proven definitively only by rechallenge. However, the assessment of cellular immunity can supplement the data on humoral response. The specificity of T-cell assays critically depends on the antigen used for stimulation. In this study, we chose the S protein as stimulus because of its importance as a target for neutralizing antibodies and because it contains major immunodominant epitopes. It mediates the entry of the SARS-CoV-2 virus into the host cell (*14*,*15*). The S1 subunit of the S protein acts on the cell binding, and the S2 subunit acts on the fusion of the viral membrane to the cell membrane (*16*; H. Wang et al., unpub. data, https://doi.org/10.1101/2020.03.26.994756). Data by Okba et al. (*17*) indicate that S1 is the most specific antigen for the diagnosis of COVID-19. The S2 subunit is the more conserved one, and could cross-react with the S protein of severe acute respiratory syndrome coronavirus (SARS-CoV-1) or MERS-CoV (*17**,**18*). However, the infection rate with SARS-CoV-1 or MERS-CoV appears low in Caucasian populations. We selected the M protein, another surface protein of SARS-CoV-2, as a second stimulus because it has been observed to also contain dominant T-cell epitopes (*19*). It plays a central role in virus assembly (*20*) and is more likely a target of cross-reactive T cells. Structural comparisons of SARS-CoV-1 and SARS-CoV-2 proteins showed 76% identity for the S protein and more for other structural proteins: 91% for the envelope protein, 90% for the M protein, and 95% for nucleocapsid (*19*). We observed that potential CP donors with undetectable antibodies against the S1 protein of SARS-CoV-2 had T-cell responses more strongly directed against the M than the S1 protein. Thus, we speculated that their T cells may preferentially target viral peptides involved in virus assembly rather than cell binding; this hypothesis needs confirmation. Responses toward the S1/S2 peptides were stronger than to the S1 protein, possibly because additional immunodominant T-cell epitopes in the S2 antigen caused the stronger response. The finding that the T-cell responses to the S1 protein, which is most specific to SARS-CoV-2, were relatively low raises the issue of potential cross-reactivity after stimulation with the S1/S2 or M peptides. Cross-reactivity has been shown for antibodies directed against SARS-CoV-1 and SARS-CoV-2 (*13*). 

Similarly, SARS-CoV-2 cross-reactive T cells due to contact to common coronaviruses may occur (*5*,*21*) that could interfere with the specificity of our ELISpot assays. Nevertheless, recent PCR-confirmed SARS-CoV-2 infection could have caused the frequency of reactive T cells toward SARS-CoV-2 to be higher in the current cohort of potential CP donors than those reactive toward other common coronaviruses. Furthermore, cross-reactive T cells could be protective against SARS-CoV-2 infection, especially in children and young adults with frequent social contacts (*5*). Using flow cytometry, Braun et al. (*5*) detected preexisting SARS-CoV-2 S-cross-reactive CD4+ T cells in 34% of healthy donors, and Grifoni et al. (*25*) in »40%–60% of unexposed persons. However, arguing against cross-reactivity interfering with our ELISpot assays, we observed negative T-cell responses in the negative control group.

Of interest, differences between participants with a ratio of <1.1 and of >3 seem to be more pronounced after stimulation with the S1 protein than the S1/S2 peptides. Thus, apart from the M protein, the S2 protein may be an additional target of T-cell responses, especially in participants with undetectable T- and B-cell responses against the S1 protein.

Chandrashekar et al. observed near-complete protection in 9 rhesus macaques after SARS-CoV-2 infection (*22*). After initial viral clearance, upon rechallenge, the animals showed a 5 log_10_ reduction in median viral loads compared with primary infection and an anamnestic humoral and cellular immune response. Moreover, Deng et al. reported that viral load remained negative in 4 rhesus macaques upon rechallenge with SARS-CoV-2 but showed a transient increase in body temperature (*23*). Similarly, Kirkcaldy et al. reported limited evidence of reinfection in humans with previously documented COVID-19 (*24*). Other studies demonstrated that SARS-CoV-2–specific T cells were detectable in the majority of recovered patients (*21*; N.L. Bert et al., unpub. data, https://doi.org/10.1101/2020.05.26.115832). Data on the earlier coronavirus SARS-CoV-1 indicated that cellular immunity was detectable for >17 years after infection. Similar to our findings, studies from Sweden and France recently observed T cell responses against SARS-CoV-2 in seronegative persons (*6*; Gallais et al.), Sekine et al. reported that 4/31 (13%) patients who recovered from mild symptoms of COVID-19 were seronegative, which is very similar to 17% of seronegative results in our cohort. Assessment of T cell immunity by flow cytometry showed a greater difference of T-cell responses toward S1/S2 and M peptide pools between seronegative and seropositive patients than our study; this difference may be attributable to several differences between the studies; that is, we here used the ELISpot method instead of flow cytometry to measure specific T cells and our seronegative CP donors were all PCR positive, whereas none of the CP donors was tested positive by SARS-CoV-2 PCR (*6*).

ELISpot data on other coronaviruses have been reported since 2004. The authors of these early studies used either human leukocyte antigens (HLA)–A2 restricted peptides (*25*) or overlapping peptide pools spanning the whole SARS-CoV-1 proteome (*26*). ELISpot data in patients in China who recovered from SARS-CoV-1 infection 1 month earlier showed T-cell immunity in 100% of participants (*25*), and in 50% of patients recovered 12 months earlier (*26*). Our ELISpot data determined at a median of 2 months after the onset of symptoms indicate that 79% of participants had detectable T-cell immunity, which fits well with the previous data on the structurally related coronavirus SARS-CoV-1. A study on SARS-CoV-1 from 2008 (*26*) showed that T cell responses were mainly directed against the S protein and that CD8+ T-cell responses were more frequent and of a greater magnitude than CD4+ T-cell responses. Furthermore, a recent study indicated that on day 14 after injection of an adenovirus-vectored COVID-19 vaccine vigorous ELISpot responses against overlapping peptides of the S protein were induced (*27*). Compared with our data, responses at day 14 were higher. However, compared with a recent study using mosaic surface protein consisting of exposed extracellular domains of the SARS-CoV-2 spike, envelope, and membrane proteins (*28*), we observed slightly stronger T-cell responses in our convalescent patients, although the assays with mosaic surface protein were performed earlier after the onset of symptoms (day 6–32). This difference could be attributable to the use of various stimuli.

As of August 2020, we face the challenge of estimating how many persons are still susceptible to SARS-CoV-2 infection. The ELISpot assay we established may help to identify patients with adaptive immunity against SARS-CoV-2 infection. The assay has the following advantages: it is applicable for routine use, measures cellular immunity within 1 day on a single cell level, determines functional cells, and is independent from HLA restriction. However, it does not allow researchers to determine which T-cell population responds upon restimulation. According to our data in volunteers with confirmed SARS-CoV-2 infection, it could be speculated that the majority of persons with undetectable systemic IgG may presumably be protected by specific T-cell immunity, which would be good news for the control of the pandemic.

Appendix Additional information about cellular immunity and SARS-CoV-2–specific IgG antibodies in convalescent COVID-19 patients.

## References

[1] Chen L, Xiong J, Bao L, Shi Y. Convalescent plasma as a potential therapy for COVID-19. Lancet Infect Dis. 2020;20:398–400. 10.1016/S1473-3099(20)30141-9 32113510 PMC7128218

[2] Shen C, Wang Z, Zhao F, Yang Y, Li J, Yuan J, et al. Treatment of 5 critically ill patients with COVID-19 with convalescent plasma. JAMA. 2020;323:1582–9. 10.1001/jama.2020.4783 32219428 PMC7101507

[3] Salazar E, Christensen PA, Graviss EA, Nguyen DT, Castillo B, Chen J, et al. Treatment of coronavirus disease 2019 patients with convalescent plasma reveals a signal of significantly decreased mortality. Am J Pathol. 2020;S0002-9440(20)30370-9. 32795424 10.1016/j.ajpath.2020.08.001 PMC7417901

[4] Zhao J, Alshukairi AN, Baharoon SA, Ahmed WA, Bokhari AA, Nehdi AM, et al. Recovery from the Middle East respiratory syndrome is associated with antibody and T-cell responses. Sci Immunol. 2017;2:eaan5393. 10.1126/sciimmunol.aan5393 28778905 PMC5576145

[5] Braun J, Loyal L, Frentsch M, Wendisch D, Georg P, Kurth F, et al. SARS-CoV-2-reactive T cells in healthy donors and patients with COVID-19. Nature. 2020. 10.1038/s41586-020-2598-9 32726801

[6] Sekine T, Perez-Potti A, Rivera-Ballesteros O, Strålin K, Gorin J-B, Olsson A, et al.; Karolinska COVID-19 Study Group. Robust T cell immunity in convalescent individuals with asymptomatic or mild COVID-19. Cell. 2020 Aug 14 [Epub ahead of print]. 10.1016/j.cell.2020.08.017 PMC7427556 32979941

[7] Heilingloh CS, Aufderhorst UW, Schipper L, Dittmer U, Witzke O, Yang D, et al. Susceptibility of SARS-CoV-2 to UV irradiation. Am J Infect Control. 2020;48:1273–5. 10.1016/j.ajic.2020.07.031 32763344 PMC7402275

[8] Liu Y, Yan LM, Wan L, Xiang TX, Le A, Liu JM, et al. Viral dynamics in mild and severe cases of COVID-19. Lancet Infect Dis. 2020;20:656–7. 10.1016/S1473-3099(20)30232-2 32199493 PMC7158902

[9] Zhou F, Yu T, Du R, Fan G, Liu Y, Liu Z, et al. Clinical course and risk factors for mortality of adult inpatients with COVID-19 in Wuhan, China: a retrospective cohort study. Lancet. 2020;395:1054–62. 10.1016/S0140-6736(20)30566-3 32171076 PMC7270627

[10] Tay MZ, Poh CM, Rénia L, MacAry PA, Ng LFP. The trinity of COVID-19: immunity, inflammation and intervention. Nat Rev Immunol. 2020;20:363–74. 10.1038/s41577-020-0311-8 32346093 PMC7187672

[11] Ni L, Ye F, Cheng ML, Feng Y, Deng YQ, Zhao H, et al. Detection of SARS-CoV-2-specific humoral and cellular immunity in COVID-19 convalescent individuals. Immunity. 2020;52:971–7 e3. 32413330 10.1016/j.immuni.2020.04.023 PMC7196424

[12] To KK, Tsang OT, Leung WS, Tam AR, Wu TC, Lung DC, et al. Temporal profiles of viral load in posterior oropharyngeal saliva samples and serum antibody responses during infection by SARS-CoV-2: an observational cohort study. Lancet Infect Dis. 2020;20:565–74. 10.1016/S1473-3099(20)30196-1 32213337 PMC7158907

[13] Hoffmann M, Kleine-Weber H, Schroeder S, Kruger N, Herrler T, Erichsen S, et al. SARS-CoV-2 cell entry depends on ACE2 and TMPRSS2 and is blocked by a clinically proven protease inhibitor. Cell. 2020;181:271–80 e8. 32142651 10.1016/j.cell.2020.02.052 PMC7102627

[14] Zhou P, Yang XL, Wang XG, Hu B, Zhang L, Zhang W, et al. A pneumonia outbreak associated with a new coronavirus of probable bat origin. Nature. 2020;579:270–3. 10.1038/s41586-020-2012-7 32015507 PMC7095418

[15] Wrapp D, Wang N, Corbett KS, Goldsmith JA, Hsieh CL, Abiona O, et al. Cryo-EM structure of the 2019-nCoV spike in the prefusion conformation. Science. 2020;367:1260–3. 10.1126/science.abb2507 32075877 PMC7164637

[16] Wang C, Li W, Drabek D, Okba NMA, van Haperen R, Osterhaus ADME, et al. A human monoclonal antibody blocking SARS-CoV-2 infection. Nat Commun. 2020;11:2251. 10.1038/s41467-020-16256-y 32366817 PMC7198537

[17] Okba NMA, Müller MA, Li W, Wang C, GeurtsvanKessel CH, Corman VM, et al. Severe acute respiratory syndrome coronavirus 2–specific antibody responses in coronavirus disease patients. Emerg Infect Dis. 2020;26:1478–88. 10.3201/eid2607.200841 32267220 PMC7323511

[18] Jaimes JA, André NM, Chappie JS, Millet JK, Whittaker GR. Phylogenetic analysis and structural modeling of SARS-CoV-2 spike protein reveals an evolutionary distinct and proteolytically sensitive activation loop. J Mol Biol. 2020;432:3309–25. 10.1016/j.jmb.2020.04.009 32320687 PMC7166309

[19] Ahmed SF, Quadeer AA, McKay MR. Preliminary identification of potential vaccine targets for the COVID-19 coronavirus (SARS-CoV-2) based on SARS-CoV immunological studies. Viruses. 2020;12:254. 10.3390/v12030254 32106567 PMC7150947

[20] Neuman BW, Kiss G, Kunding AH, Bhella D, Baksh MF, Connelly S, et al. A structural analysis of M protein in coronavirus assembly and morphology. J Struct Biol. 2011;174:11–22. 10.1016/j.jsb.2010.11.021 21130884 PMC4486061

[21] Grifoni A, Weiskopf D, Ramirez SI, Mateus J, Dan JM, Moderbacher CR, et al. Targets of T cell responses to SARS-CoV-2 coronavirus in humans with COVID-19 disease and unexposed individuals. Cell. 2020;181:1489–501. 32473127 10.1016/j.cell.2020.05.015 PMC7237901

[22] Chandrashekar A, Liu J, Martinot AJ, McMahan K, Mercado NB, Peter L, et al. SARS-CoV-2 infection protects against rechallenge in rhesus macaques. Science. 2020;369:812–7. 10.1126/science.abc4776 32434946 PMC7243369

[23] Deng W, Bao L, Liu J, Xiao C, Liu J, Xue J, et al. Primary exposure to SARS-CoV-2 protects against reinfection in rhesus macaques. Science. 2020;369:818–23. 10.1126/science.abc5343 32616673 PMC7402625

[24] Kirkcaldy RD, King BA, Brooks JT. COVID-19 and postinfection immunity. JAMA. 2020;323:2245–6. 10.1001/jama.2020.7869 32391855 PMC8596300

[25] Wang YD, Sin WY, Xu GB, Yang HH, Wong TY, Pang XW, et al. T-cell epitopes in severe acute respiratory syndrome (SARS) coronavirus spike protein elicit a specific T-cell immune response in patients who recover from SARS. J Virol. 2004;78:5612–8. 10.1128/JVI.78.11.5612-5618.2004 15140958 PMC415819

[26] Li CK, Wu H, Yan H, Ma S, Wang L, Zhang M, et al. T cell responses to whole SARS coronavirus in humans. J Immunol. 2008;181:5490–500. 10.4049/jimmunol.181.8.5490 18832706 PMC2683413

[27] Zhu FC, Li YH, Guan XH, Hou LH, Wang WJ, Li JX, et al. Safety, tolerability, and immunogenicity of a recombinant adenovirus type-5 vectored COVID-19 vaccine: a dose-escalation, open-label, non-randomised, first-in-human trial. Lancet. 2020;395:1845–54. 10.1016/S0140-6736(20)31208-3 32450106 PMC7255193

[28] Thijsen S, Heron M, Gremmels H, van der Kieft R, Reusken C, Kremer K, et al. Elevated nucleoprotein-induced interferon-γ release in COVID-19 patients detected in a SARS-CoV-2 enzyme-linked immunosorbent spot assay. J Infect. 2020;81:452–82. 10.1016/j.jinf.2020.06.015 32540458 PMC7290187

