# Supplementary material for: Cellular Immunity in COVID-19 Convalescents with PCR-Confirmed Infection but with Undetectable SARS-CoV-2–Specific IgG
Source: Emerg Infect Dis. 2021 Jan;27(1):122–9. doi: 10.3201/eid2701.203772 (PMC7774536; doi:10.3201/eid2701.203772)
Supplement: Appendix — Additional information about cellular immunity and SARS-CoV-2–specific IgG antibodies in convalescent COVID-19 patients. [file 20-3772-Techapp-s1.pdf]

Article DOI: https://doi.org/10.3201/eid2701.203772

# Cellular Immunity in COVID-19 Convalescents with PCR-Confirmed Infection but with Undetectable SARS-CoV-2–Specific IgG

## Appendix

### Cutoff Definition for the SARS-CoV-2–specific ELISpot

The cutoff was based on the negative control values (nonstimulated cultures) and on the consideration that 3-fold higher values for stimulated versus nonstimulated cells are frequently considered as a positive response in cellular assays. The negative controls had on average 0.27 spots (range 0–2) and its 3-fold standard deviation was 3 × 0.48 spots = 1.44 spots, which we considered as background of the negative controls. As we used increment values, a 3-fold higher value versus background means 3 × 1.44 spots minus 1 × 1.44 spots, i.e., 2.88 spots increment. We therefore chose 3 as cutoff for positivity, together with the criterion of 3-fold higher values of stimulated versus non-stimulated cultures (which was fulfilled in all cases).

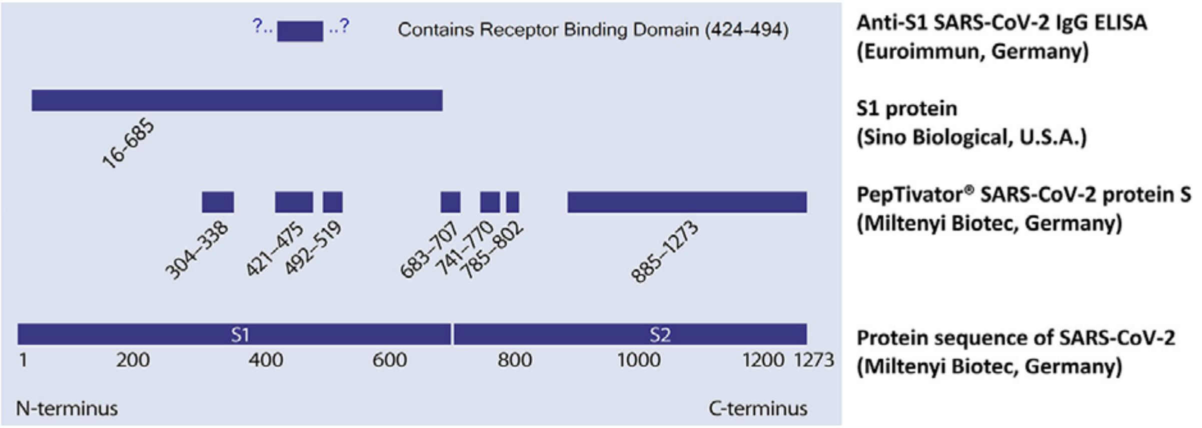


**Appendix Figure 1.** Sequence alignment of the S antigens of SARS-CoV-2. This figure contains all available information on the S antigens used for the ELISA and ELISpot assays. We modified an image

that Miltenyi Biotec shows on its homepage (https://www.miltenyibiotec.com/DE-en/products/peptivator-sars-cov-2-prot-s-107090.html#130-126-700), containing information on their PepTivator product and the protein sequence of SARS-CoV.2. The peptide mix (PepTivator) of the S protein (S1/S2) consists mainly of 15-mer sequences with 11 amino acids overlap, covering the immunodominant sequence domains of the surface glycoprotein of SARS-CoV-2. The S1 protein (Sino Biological, http://www.sinobiological.com) is a recombinant protein expressed in (human) HEK293 cells. The S1 antigen used in the ELISA assay (Euroimmun, https://www.euroimmun.com) is also produced in HEK293 cells and contains the S1 domain including the immunologically relevant receptor binding domain.

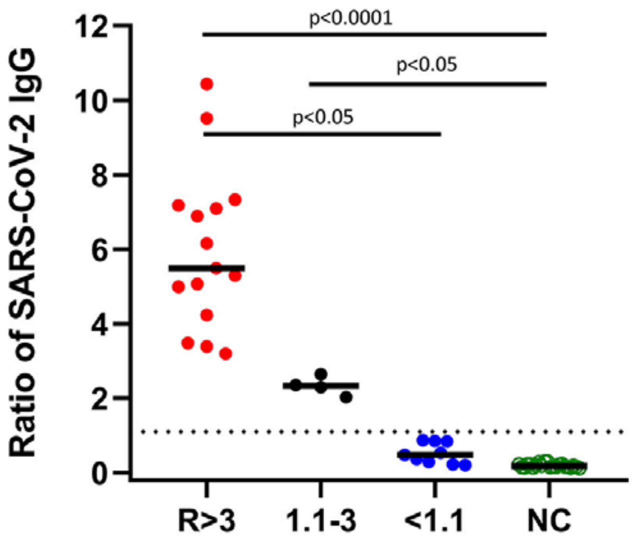


**Appendix Figure 2.** Antibody results determined parallel to cellular immunity against SARS-CoV-2 (Figure 2). The 3 left groups represent potential convalescent plasma donors with PCR-confirmed SARS-CoV-2 infection. They either had a strong positive antibody response to an S1-specific Anti-SARS-CoV-2 IgG ELISA (Euroimmun) as defined by an antibody ratio (R) of >3 (n = 15); an intermediate response (ratio of 1.1-3, n = 4); or borderline or negative results (ratio of <1.1, n = 9). The right group displays data in healthy controls without symptoms of respiratory or gastrointestinal infections and without household contact with SARS-CoV-2 infected patients since January 2020 (negative controls, NC, n = 22). The latter group has been tested negative or has not been tested by SARS-CoV-2 PCR. Responses in the 4 groups of volunteers were compared by Kruskal-Wallis test with Dunn’s correction. The dotted line represents a ratio of 1.1, the cutoff for positive antibody responses.

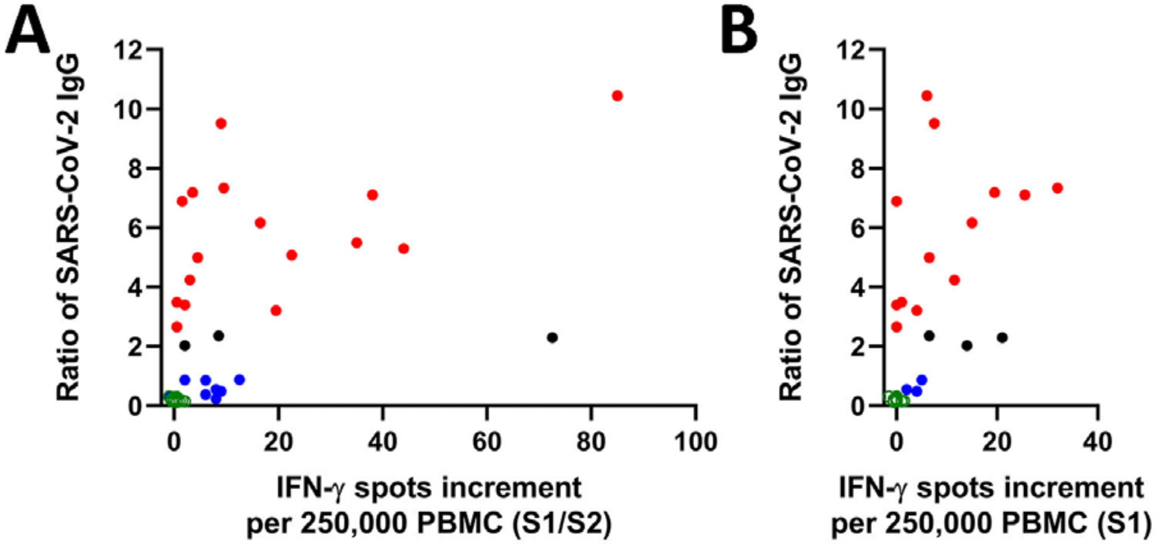


**Appendix Figure 3.** Interrelationship between cellular and humoral immunity to S antigens of SARS-CoV-2. The plots include the first data set in potential convalescent-plasma donors and in negative controls. We used the following color-coding: antibody ratio to an S1-specific Anti-SARS-CoV-2 IgG ELISA (Euroimmun) >3: red; 1.1-3: black; <1.1: blue; negative controls: green. The left panel shows ELISpot data on a peptide pool of the S1/S2 antigen and the right on an S1 protein antigen of SARS-CoV-2.
